# Prion-Dependent Lethality of *sup35* Missense Mutations Is Caused by Low GTPase Activity of the Mutant eRF3 Protein

**DOI:** 10.3390/ijms26073434

**Published:** 2025-04-06

**Authors:** Nina P. Trubitsina, Olga M. Zemlyanko, Andrew G. Matveenko, Stanislav A. Bondarev, Svetlana E. Moskalenko, Evgeniia M. Maksiutenko, Anna A. Zudilova, Tatiana M. Rogoza, Galina A. Zhouravleva

**Affiliations:** 1 Department of Genetics and Biotechnology, St. Petersburg State University, 199034 St. Petersburg, Russia; n.trubitsina@spbu.ru (N.P.T.); o.zemlyanko@spbu.ru (O.M.Z.); a.matveenko@spbu.ru (A.G.M.); s.bondarev@spbu.ru (S.A.B.); s.moskalenko@spbu.ru (S.E.M.); evgeniia_maksiutenko@mail.ru (E.M.M.); aniuta.zudilova@gmail.com (A.A.Z.); t.rogoza@spbu.ru (T.M.R.); 2 Laboratory of Amyloid Biology, St. Petersburg State University, 199034 St. Petersburg, Russia; 3 St. Petersburg Branch, Vavilov Institute of General Genetics, Russian Academy of Sciences, 199034 St. Petersburg, Russia

**Keywords:** prion, [*PSI*^+^], translation termination, Sup35, missense mutations, eRF3, GSPT1, GTPase

## Abstract

The essential *SUP35* gene encodes yeast translation termination factor Sup35/eRF3. The N-terminal domain of Sup35 is also responsible for Sup35 prionization that leads to generation of the [*PSI*^+^] prion. Previously we isolated different types of *sup35* mutations (missense and nonsense) and demonstrated that *sup35* nonsense mutations (*sup35-n*) are incompatible with the [*PSI*^+^] prion, leading to lethality of *sup35-n* [*PSI*^+^] haploid cells. Here, we show that *sup35* missense mutations (*sup35-m*) within conservative regions of the Sup35 C-domain result in lethality of [*PSI*^+^] cells because of weak activity of Sup35/eRF3 as a translation termination factor. Mutant Sup35 maintain their ability to be incorporated into pre-existing [*PSI*^+^] aggregates and to form amyloid aggregates in vitro, while *sup35-m* mutations do not influence the [*PSI*^+^] prion induction and stability. All these mutations (D363N, R372K, T378I) are located in the conservative GTPase region of Sup35, decreasing the GTPase activity of mutated proteins. We propose that such low activity of mutant Sup35 combined with aggregation of Sup35 constituting the [*PSI*^+^] prion is not sufficient to maintain the viability of yeast cells.

## 1. Introduction

Translation termination in eukaryotes is regulated by two release factors, eRF1 and eRF3, encoded in yeast by the *SUP45* and *SUP35* gene, respectively [1,2]. The Sup35 protein consists of three domains: N-terminal (Sup35N), M (middle, Sup35M), and C-terminal (Sup35C). While Sup35N is the prion-forming domain, Sup35M is involved in phase separation and interaction with chaperones, and Sup35C is the catalytic domain responsible for translation termination (reviewed in [3]). Only the C-terminal domain is conserved among different eukaryotes, which may be explained by its important function in translation termination. It is in this domain that the sites of interaction with eRF1, as well as the GTPase sites, are located (reviewed in [4]). A polymorphism was detected between the *SUP35* gene sequence of the Peterhof Genetic Collection (PGC) lineage and the reference sequence presented in GenBank (strain S288C). All polymorphic sites were located in the non-conserved N and M domains and did not have their own phenotypic manifestation [5]. The Sup35N domain is responsible for the prionization of Sup35, which leads to the formation of the [*PSI*^+^] prion; the prion cannot exist in its absence (for a review, see [6]). Sup35 aggregation in [*PSI*^+^] cells results in defective translation termination, leading to an omnipotent nonsense suppression. Most of the mutations leading to [*PSI*^+^] destabilization, prion loss, or problems with prion induction or maintenance described so far have been located in the Sup35N (reviewed in [7]). In contrast, mutations affecting the activity of Sup35 as a translation termination factor, eRF3, have been mapped to the C-terminal portion of this protein (reviewed in [4]).

Previously we isolated different types of *sup35* mutations and demonstrated that *sup35* nonsense mutations (*sup35-n*) are incompatible with the [*PSI*^+^] prion, leading to lethality of *sup35-n* [*PSI*^+^] haploid cells. In diploid cells, compatibility of [*PSI*^+^] with *sup35-n* depends on how the corresponding diploid was obtained, suggesting that cells with additional mutations or amplification of the mutant gene may be selected during the cultivation of [*psi*^−^] *sup35-n* haploids [8,9]. Here, we continue this line of research with the aim of answering the following question: Are the *sup35* missense mutations (*sup35-m*) located in the conserved GTPase region of Sup35 compatible with the [*PSI*^+^] prion? Our data show that, similarly to the previously studied *sup45* mutations, *sup35-m* mutations are incompatible with prion formation. This phenomenon can be explained by the reduced GTPase activity of the translation termination factor eRF3 in *sup35-m* mutants, which, in combination with already aggregated Sup35 in [*PSI*^+^] cells, is insufficient to maintain the viability of yeast cells.

## 2. Results

### 2.1. Strain-Specific Mutations in the Middle (M) Domain of Sup35 Do Not Affect the Manifestation of Missense Mutations in the C-Terminal Domain of Sup35

**Figure 1:**
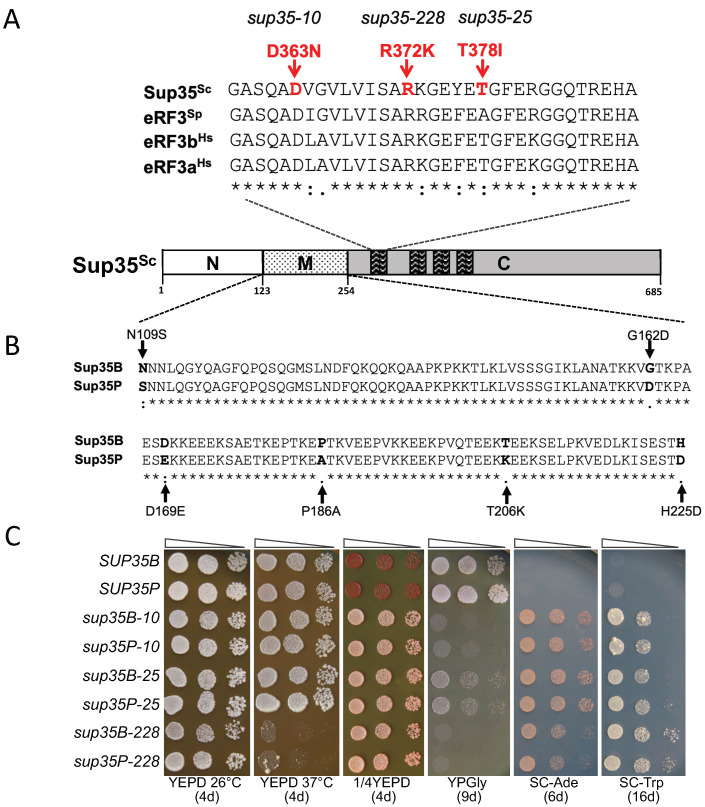
Missense mutations of the *SUP35* gene studied in this work. (**A**) The location of missense mutations in the C-domain of Sup35 (indicated in red). Multiple sequence alignment of *S. cerevisiae* Sup35 (aa 358–390), *S. pombe* eRF3 (aa 336–370), *H. sapiens* eRF3b (aa 301–333), and eRF3a (aa 310–342); only the part of the alignment containing *sup35* mutations in the Clustal format is demonstrated. The symbols (*), (:), and (.) indicate positions in the alignment that have fully, moderately, and weakly conserved residues, respectively. (**B**) The location of amino acid substitutions in the M-domain of Sup35 (aa 109–225) in the reference strain S228C from GenBank (designated as Sup35B) and strains of the PGC (designated as Sup35P). Domains are shown out of scale. (**C**) Plate assay showing the growth of yeast strains, derived from U-14-D1690-bearing plasmids with missense mutations in the *SUP35* gene, on the synthetic medium without adenine (SC-Ade), or tryptophan (SC-Trp), at 26 °C. indicating the suppression of *ade1-14* and *trp1-289*, respectively. Thermosensitivity (growth at 37 °C), color phenotype (1/4YEPD) and growth on non-fermentable carbon source (YPGly) were also assessed; number of days of incubation for each plate is indicated below in parentheses. Tenfold serial dilutions of yeast suspensions of the same density were used. Five independent clones were tested; representative results are shown.

Three mutant alleles in the *SUP35* gene described previously were used in this work. All these mutations are localized in the conservative part of the C-terminal domain of the Sup35 protein (Figure 1A). The mutations were selected on different genotypic backgrounds. One of them, *sup35-228* (R372K), was obtained by screening for simultaneous reversion of *ade1-14* (UGA) and *his7-1* (UAA) phenotypes in the 1B-D1606 strain [10,11]. This strain contains the reference *SUP35* allele described in GenBank (https://www.ncbi.nlm.nih.gov/datasets/genome/GCF_000146045.2 (accessed on 4 March 2025)). Mutations *sup35-10* (D363N) and *sup35-25* (T378I) were isolated by simultaneous suppression of the *ade1-14*, *his7-1*, and *lys2-87* (UGA) nonsense mutations in the 2V-P3982 strain [12]. The 2V-P3982 strain contains the *SUP35* allele from PGC (Peterhof Genetic Collection). Hereinafter, *SUP35* alleles will be designated as *SUP35P* and *SUP35B* for the Peterhof and GenBank alleles, respectively. The sequences of both alleles differed by 10 nucleotides, leading to six missense mutations in the central part of the *SUP35* gene (four other nucleotide substitutions were synonymous) [5] (Figure 1B).

To find out whether the substitutions in the Peterhof allele of *SUP35* affect the phenotype of missense mutations in the C-domain, we cloned the *sup35-10* and *sup35-25* mutations into the reference *SUP35* allele on the centromeric plasmid, and cloned the *sup35-228* mutation into the Peterhof allele. Using plasmid shuffling, the strains were obtained which contained the mutant alleles as an only copy of the *SUP35* gene. The phenotypes of the resulting strains were then compared (Figure 1C). No significant phenotypic differences could be observed between the strains carrying missense mutant alleles on the Peterhof or reference background.

### 2.2. Missense Mutations in the C-Terminal Domain of Sup35 Are Incompatible with the [*PSI*^+^] Prion

**Figure 2:**
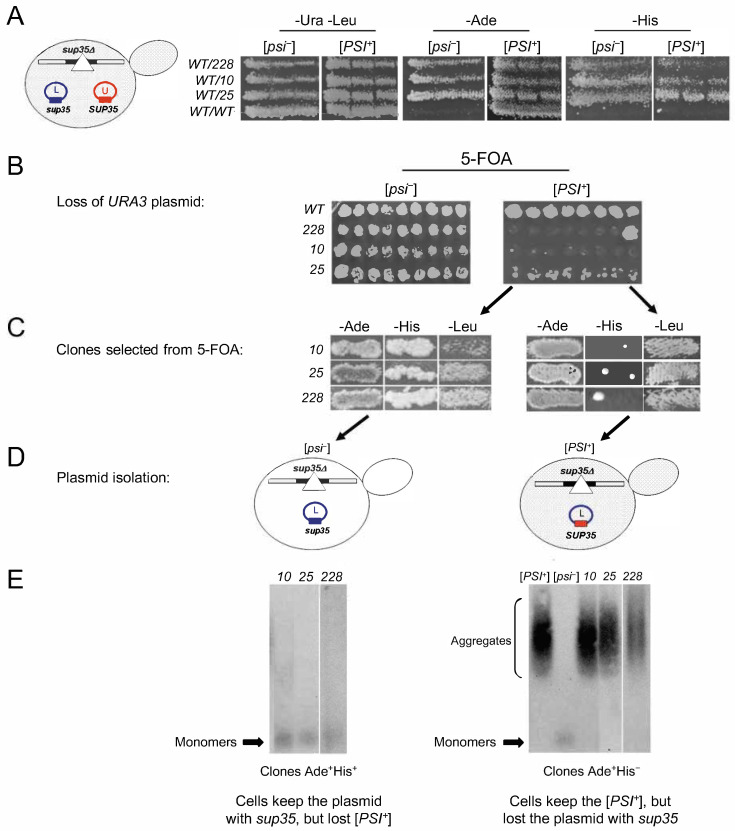
Missense mutations in the C-terminal domain of Sup35 are incompatible with the [*PSI*^+^] prion. Alleles of the *SUP35* gene designated on the panels as *WT*, *10*, *25*, and *228* correspond to *SUP35B*, *sup35P-10*, *sup35P-25*, and *sup35B-228*, respectively. The *URE3* plasmid (U) and the *SUP35* wild-type allele are marked in red, while the *LEU2* plasmid (L) and the mutant *sup35* allele are marked in blue. (**A**) Strains 7A-D832 [*psi*^−^] and 10-7A-D832 [*PSI*^+^] contain two plasmids: the first, with the wild-type *SUP35B*, and the second, with *sup35-m*. (**B**) Strains shown on panel A, were replica-plated on 5-FOA medium to select against the *SUP35WT* plasmid. Nine independent transformants are shown in each case. (**C**) Clones obtained after selection on 5-FOA differ by the ability to suppress the *his7-1* mutation. Panels represent the phenotype of clones selected from 5-FOA where [*PSI*^+^] cells were plated. (**D**) The plasmids were purified and tested by restriction or sequencing from strains shown on panel C. (**E**) Yeast cell lysates from transformants shown on panel C were characterized by SDD-AGE followed by immunoblotting with anti-Sup35 antibodies.

To test whether the *sup35* missense mutations are able to maintain yeast cell viability in the presence of the [*PSI*^+^] prion, we transformed strains 10-7A-D832 [*PSI*^+^] and 7A-D832 [*psi*^−^] with plasmids bearing either *SUP35WT* or the *sup35-m* (*sup35-10*, *sup35-25*, *sup35-228*) mutant allele. When plated onto selective media containing leucine and uracil and thus allowing for the loss of plasmid with either *SUP35WT* or *sup35-m*, all the [*psi*^−^] strains with a mutant allele demonstrated a strong suppressor phenotype (Ade^+^His^+^), while in the [*PSI*^+^] strains, the suppression was weaker for the *sup35-228* and *sup35-10* mutations (Figure 2A). As [*PSI*^+^] itself is unable to suppress *his7-1*, these results may indicate that the [*PSI*^+^] cells preferentially retain *SUP35WT* and lose the mutant alleles.

To select against the *SUP35WT* plasmid, the transformed cells were grown on 5-FOA medium (Figure 2B). While the [*psi*^−^] strains were able to efficiently lose the *SUP35WT* plasmid, the efficiency of loss in the [*PSI*^+^] strains depended on the *sup35-m* mutation (Figure 2B). Presumably, [*PSI*^+^] clones selected from the 5-FOA medium were tested by growth on selective media (Figure 2C). All of them were Leu^+^, which confirms the presence of a *LEU2* plasmid and Ade^+^, which may indicate the presence of *sup35-m* mutation or/and prion [*PSI*^+^]. However, most of these clones (90%) were His^−^ and few of them (10%) kept the His^+^ phenotype. To explain this discrepancy, we tested for the type of the plasmid and for presence of the [*PSI*^+^] aggregates in the transformants selected from 5-FOA. Plasmids were purified from these transformants and verified by restriction (for *sup35-10* and *sup35-25*) or sequencing (for *sup35-228*) (Figure 2D). Clones Ade^+^His^+^ (Figure 2C, left panel) contained the *sup35-m LEU2* plasmid, while in clones Ade^+^His^−^ (Figure 2C, right panel), the wild-type *SUP35* plasmid was present. In accordance with these data, the SDD-AGE method showed that rare Ade^+^His^+^ clones do not contain [*PSI*^+^] prion aggregates (Figure 2E, left panel), while the Ade^+^His^−^ clones still keep the [*PSI*^+^] prion (Figure 2E, right panel). From these data, we can conclude that the [*PSI*^+^] prion is maintained only in the presence of the *SUP35WT* allele.

### 2.3. Missense Mutations in the C-Terminal Domain of Sup35 Do Not Influence the [*PSI*^+^] Prion Induction and Stability

**Figure 3:**
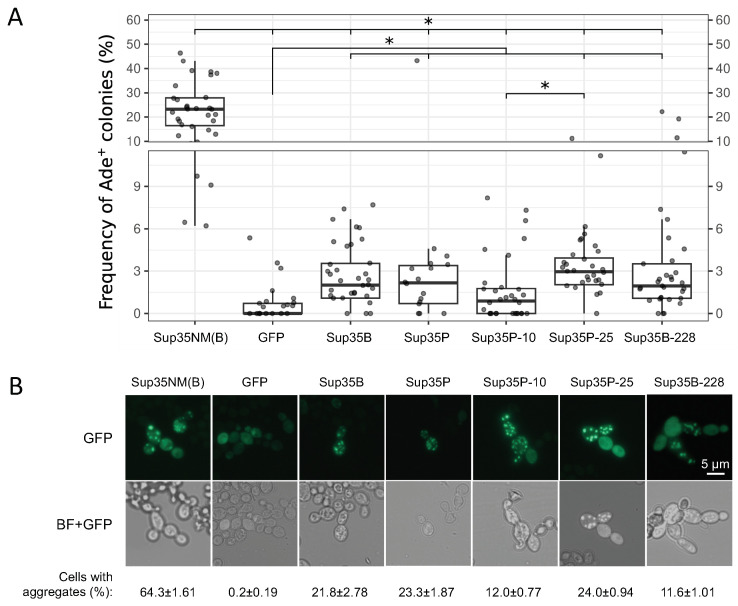
[*PSI*^+^] induction by mutant *sup35-m* alleles. The 7A-D832 [*pin*^−^][*PIN*^+^] strain was transformed with pRS316-pCUP-GFP, pRS316-pCUP-SUP35NM-GFP, pRS316-pCUP-SUP35-GFP or pRS316-pCUP-sup35-m-GFP plasmids. Logarithmic yeast cultures were incubated in the presence of 50 μM CuSO_4_ for 24 h. (**A**) Comparison of the frequency of appearance of [*PSI*^+^] clones after the induction of constructs with different *SUP35* alleles. Statistical processing of data was carried out using the Wilcoxon rank-sum test adjusted for multiple comparisons (Holm’s method). *, *p* < 0.05. (**B**) Aggregates of Sup35-GFP, as well as proteins with substitutions, do not differ in their morphology. Cells were visualized using fluorescent microscopy. Representative groups of cells are shown. Scale bar corresponds to 5 μm. BF + GFP—bright field merged with fluorescence. Below, the proportion of cells with aggregates is shown.

To estimate the efficiency of [*PSI*^+^] induction by mutant variants of Sup35 compared to full-length wild-type Sup35, strain 7A-D832 [*psi*^−^][*PIN*^+^] was transformed with a series of plasmids, in which the sequences of wild-type *SUP35* or missense mutant alleles are fused to the GFP sequence and regulated by the copper-inducible *CUP1* promoter. Two wild-type alleles were used, *SUP35B* and *SUP35P*. Transformants carrying only GFP on the plasmid were used as negative controls. The Ade^+^ phenotype in these cells occurred with a frequency of about 0.2% (Figure 3A). The frequency of [*PSI*^+^] induction by mutant alleles did not show any consistent differences between the alleles of different genetic lines. It should be noted that the frequency of [*PSI*^+^] induction by full-length Sup35 variants is very low compared to Sup35NM (Figure 3A). The presence of aggregates was detected using fluorescence microscopy. All transformants overproducing Sup35-GFP formed similar large multiple fluorescent foci (Figure 3B). However, the proportions of cells with aggregates differed insignificantly. In the presence of the *sup35-10* and *sup35-228* mutations, the number of cells with aggregates was lower, while the *sup35-25* allele had no effect. At first glance, these data do not fully agree well with the effectiveness of [*PSI*^+^] induction (Figure 3A). It is possible that the studied mutations also disrupt the interaction of Sup35 with other yeast proteins, which is reflected in the frequencies of the appearance of cells with the prion. Another explanation may be that some cells with a nonsense suppressor phenotype may not carry a prion.

### 2.4. The Mutant Sup35 Protein Is Capable of Being Incorporated into Pre-Existing [*PSI*^+^] Aggregates

**Figure 4:**
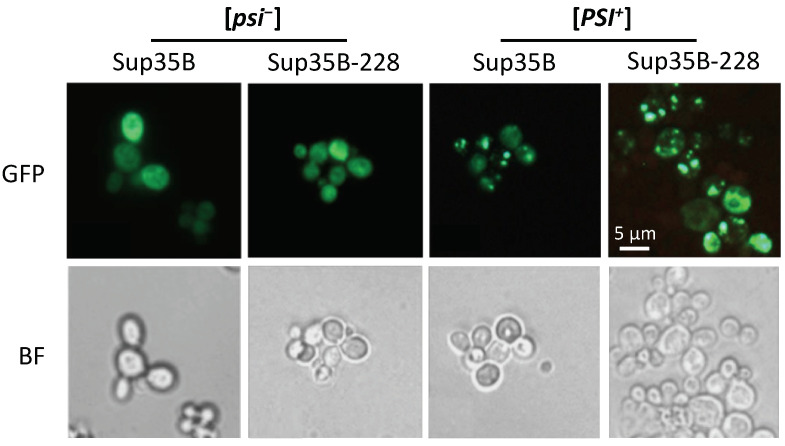
Mutant protein Sup35-228 is included to pre-existing [*PSI*^+^] aggregates. The 10-7A-D832 [*PSI*^+^] and 7A-D832 [*psi*^−^] strains were transformed with pRS316-pCUP-SUP35-GFP or pRS316-pCUP-sup35-228-GFP plasmids. Logarithmic yeast cultures were incubated in the presence of 50 μM CuSO_4_ for two hours. Cells were visualized using fluorescent microscopy. Representative groups of cells are shown. Scale bar corresponds to 5 μm. BF—bright field.

To find out whether missense mutations in the C-domain could impair the ability of mutant Sup35 to be incorporated into pre-existing [*PSI*^+^] aggregates, we analyzed their intracellular distribution after brief transient overproduction. [*PSI*^+^] and [*psi*^−^] yeast cells transformed with pRS316-pCUP-SUP35-GFP or pRS316-pCUP-sup35-228-GFP plasmid were grown in selective medium to the middle logarithmic phase. At 2 h after the construct induction by 50 μM CuSO_4_ addition, the fluorescence pattern was assessed. It is important to note that induction within 2 h does not result in de novo prion generation [13]. In [*psi*^−^] cells, as expected, mainly diffuse fluorescence was observed, both in the presence of the wild-type and mutant allele. In the [*PSI*^+^] cells carrying the *SUP35-GFP* construct, aggregates were detected in more than 50% of cells; a similar situation was observed for the mutant allele (Figure 4). This suggests that the mutant protein may be incorporated into pre-existing [*PSI*^+^] aggregates.

### 2.5. Mutant Sup35 Proteins Form Amyloid Aggregates in an In Vitro System

In Section 2.3, we demonstrated that the Sup35-m proteins can induce [*PSI*^+^] formation when the corresponding *sup35-m* allele is overexpressed in the presence of wild-type Sup35. For the Sup35-228 protein, we showed the ability to be incorporated into pre-existing [*PSI*^+^] aggregates (Section 2.4). Next, we performed in vitro aggregation experiments of the mutant proteins to test their ability to form aggregates on their own. For that, we purified recombinant Sup35-m proteins under non-denaturating conditions that were used to assemble the fibrils.

**Figure 5:**
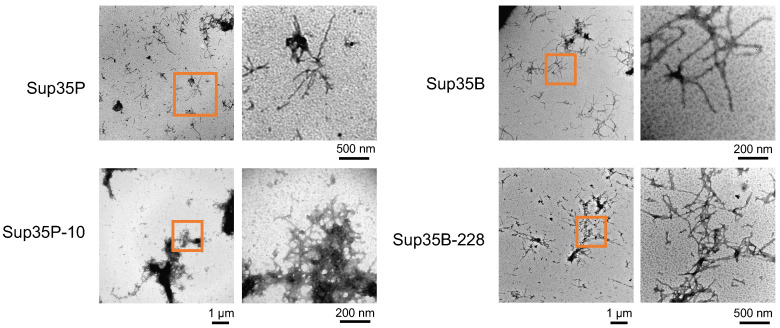
All analyzed Sup35 variants are able to form fibrils in vitro. Electron micrographs of Sup35 polymers negatively stained with uranyl acetate. Fibrils were formed in vitro by full-length Sup35 proteins (Sup35P, Sup35P-10, Sup35B, Sup35B-228). Left micrographs in each panel represent the overall image at 40,000× magnification (scale bar corresponds to 1 μm). Right micrographs were obtained by enlarging the selected area from the left images at 200,000× (Sup35P-10 and Sup35B-228), and 80,000× (Sup35P and Sup35B) magnification (scale bars correspond to 200 and 500 nm, respectively).

**Figure A1:**
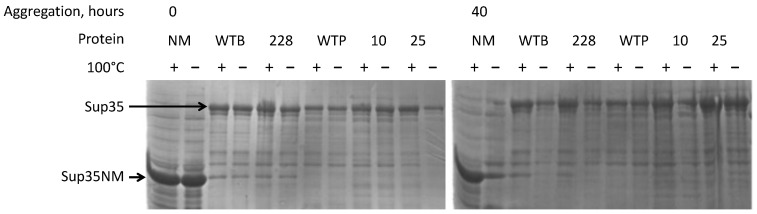
Mutant Sup35 proteins are able to form insoluble aggregates in the presence of pre-existing fibrils. Boiled and unboiled samples before (0 h) and after (40 h) Sup35 aggregation were analyzed by SDS-PAGE followed by Coomassie gel staining. Sup35 proteins designated on the panels as NM, WTB, 228, WTP, 10, and 25 correspond to Sup35NM(B), Sup35B, Sup35B-228, Sup35P, Sup35P-10, and Sup35P-25, respectively. Full-length Sup35 and Sup35NM are shown by arrows.

All the proteins studied were able to form insoluble aggregates in the presence of pre-existing fibrils, which was verified using SDS-PAGE (Figure A1). Analysis of the Sup35 aggregates was performed using transmission electron microscopy (Figure 5). For the proteins Sup35B, Sup35B-228, and Sup35P, the formation of characteristic fibrils was observed. The shape and size of these fibrils were similar. The Sup35P-10 protein formed structures that were more amorphous compared to other proteins, but nevertheless, the image shows structures similar to the fibrils of the wild-type proteins and Sup35B-228. It should also be noted that the aggregates we obtained were similar to those described in the literature, for example, in [14,15]. In these experiments only mutant Sup35B-228 and Sup35P-10 were examined, but our previous studies showed that Sup35P-25 forms fibrils similar to Sup35B-228. Thus, all full-length proteins are capable of forming fibrils; amino acid substitutions in the C-domain of proteins do not prevent fibril formation.

### 2.6. Sup35-m Mutant Proteins Demonstrate Decreased Translation Termination Factor Activity

**Figure 6:**
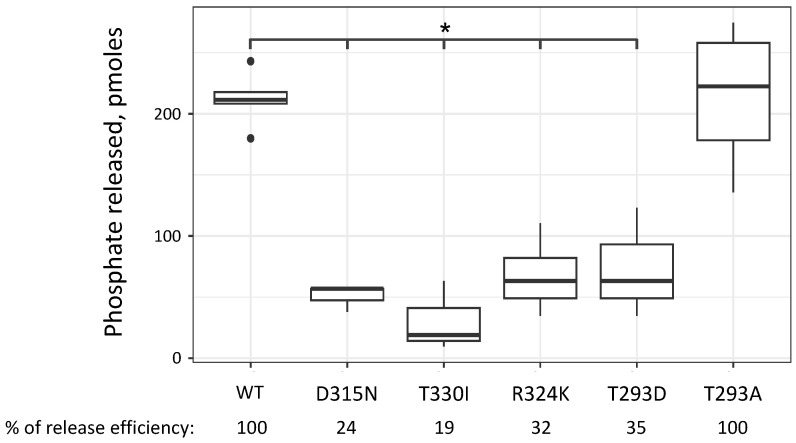
The studied missense mutations lead to a decrease in the functional activity of the eRF3a factor. GTPase activity of human eRF3a proteins was measured in the presence of the 80S ribosomes and human eRF1. The difference between the amount of phosphate released for WT and mutants (except T293A) in the Welch’s *t*-test (except D315N) and the Wilcoxon rank-sum test (for D315N) adjusted for multiple comparisons (Benjamini-Hochberg correction) was found. *, *p* < 0.05. The black dots on the graph represent outliers. Positions of mutations in human eRF3a correspond to the following positions in yeast Sup35: D315 to D363; T330 to T378; R324 to R372; T293 to T341, respectively.

During translation termination, after the eRF1-eRF3 complex binds to the stop codon on the ribosome, eRF3 hydrolyzes GTP. This is followed by a conformational change in the translation complex and positioning of eRF1 in the peptidyl transferase center of the ribosome [16]. Since the studied missense mutations do not affect prion properties, we hypothesized that lethality of mutant haploid cells [*PSI*^+^] may be associated with a decrease in the functional activity of the eRF3 factor. To test this hypothesis, we performed a GTPase assay with full-length human eRF3a proteins. Full-length yeast proteins were not suitable for this assay, since they aggregate during purification under native conditions. We purified recombinant human eRF3 proteins with the D315N, T330I, and R324K mutations, which correspond to the D363N (*sup35-10*), T378I (*sup35-25*), and R372K (*sup35-228*) substitutions in yeast Sup35, respectively. Additionally, we introduced substitutions homologous to T341A and T341D in yeast Sup35, as additional controls, since the T341D mutation was shown to compromise cell viability, while T341A had no such effect [17]. Position 341 in yeast Sup35 corresponds to the position 293 in human eRF3a. We found that all proteins with the substitutions, except for the T293A, had reduced functional activity (Figure 6). However, it was previously shown that yeast Sup35 proteins with T341A and T341D substitutions demonstrate a large decrease in GTPase activity [17]. It is possible that in human eRF3, the substitution T293A, homologous to the yeast T341A, does not lead to a decrease in functional activity. Thus, the lethality of haploid [*PSI*^+^] cells carrying only the *sup35-10*, *sup35-25*, and *sup35-228* missense mutant alleles may occur due to a decrease in the GTPase activity of the eRF3.

## 3. Discussion

In eukaryotes, termination of translation requires two release factors: eRF1 and eRF3. While the eRF1 protein belongs to class I translation termination factors responsible for the stop codon recognition and peptidyl-tRNA hydrolysis, eRF3 is a class II termination factor that functions to stimulate class I factors through its GTPase activity. Eukaryotic organisms have one class I translation termination factor called eRF1 [18] that recognizes all three stop codons. eRF3 proteins [1,2] have only one homolog in yeast *S. cerevisiae*, called Sup35, and two paralogs in placental mammals, eRF3a and eRF3b. The eRF3 proteins of most eukaryotes, with the exception of some protozoa, consist of several domains. Yeast Sup35 protein is traditionally divided into three domains: N, M, and C, where N-M and M-C domain boundaries were assigned to the second and third methionine residues, respectively. The C-terminal region of the eRF3 family proteins is highly conserved and has significant similarity to the elongation factor EF1A. Unlike the C-terminal portion, the N and M regions of the eRF3 proteins are not conserved (see review by [19]).

In *S. cerevisiae* and other budding yeast, the Sup35N (also called PFD—prion-forming domain) is responsible for the formation of the [*PSI*^+^] prion. In [*PSI*^+^] cells (but not in [*psi*^−^]), Sup35 forms protease-resistant aggregates, which results in impaired translation termination and nonsense suppression due to a decreased amount of functional Sup35 (for review, see [6]). The Sup35M region is enriched with charged amino acids and is not required for viability and translation termination but is involved in the interaction with Hsp104 [20] and other cellular factors involved in the control of the formation and propagation of the [*PSI*^+^] prion. Many of the chaperones and different factors required for the propagation of yeast prions have been described and discussed in recent reviews [21,22,23]. NM-domain forms reversible pH-dependent biomolecular condensates [24]. The role of the C-terminal domain of Sup35 for condensate formation in vivo remains contradictory [25,26].

**Figure A2:**
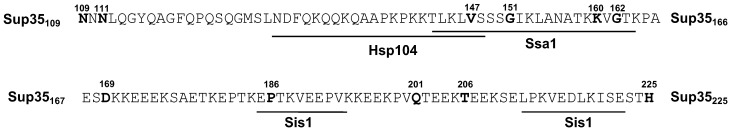
Polymorphic amino acids identified in the NM-region of Sup35 (aa 109–225) in different strains of *S. cerevisiae* (data from [5,27,28] and https://www.yeastgenome.org/locus/S000002579/sequence (accessed on 4 March 2025)) are shown in bold. Ssa1 and Sis1 binding sites in Sup35NM monomer were detected by NMR [57]. Hsp104 interaction site (aa 129–148) was determined using peptide array [20].

Analysis of clinical isolates and yeast strains of various origins revealed 11 polymorphic sites in the NM region of Sup35 (aa 109–225) [5,27,28] and https://www.yeastgenome.org/locus/S000002579/sequence (accessed on 4 March 2025)) (Figure A2). Only one of these sites is localized in Sup35-PFD (residue 109), and it was non-essential for [*PSI*^+^] propagation [29]. In our work, the *SUP35B* and *SUP35P* alleles did not differ in [*PSI*^+^] induction, forming aggregates of similar morphology (Figure 3). Interestingly, four polymorphic sites are localized in the Ssa1-interacting region of Sup35, but their influence on this interaction remains unknown. From these data, it can be concluded that all mutations affecting the [*PSI*^+^] prion are localized in the Sup35N domain—mutations in Sup35M that affect the properties of the [*PSI*^+^] are unknown. One mutation in the C-domain of Sup35 that influenced [*PSI*^+^] propagation has also been described [30]. All mutations affecting the activity of Sup35 as a translation termination factor, eRF3, are located in the C-terminal portion of this protein (reviewed in [4]).

Although the *SUP45* and *SUP35* genes are essential for yeast cells, previously, we have isolated a series of spontaneous nonsense mutations designated as *sup45-n* and *sup35-n*, respectively [10,31], and have shown that these mutations are incompatible with the [*PSI*^+^] prion in haploid cells [8,32]. In the case of *SUP45* missense mutations (*sup45-m*), viability depended on the prion type (strong or weak) and the mutant allele: while all *sup45-m* were synthetically lethal in combination with the strong [*PSI*^+^]^*S*^ prion variant, two weak nonsense suppressor alleles (*sup45-113* and *sup45-115*) were non-lethal in the presence of the weak [*PSI*^+^]^*W*^ prion variant [32]. Since the viability of the spontaneous *sup35-m* mutations in association with the [*PSI*^+^] prion has not yet been characterized, we aimed to answer this question in this study.

For these purposes, we took three spontaneous *sup35-m* mutations (D363N, R372K, T378I) located in the conserved GTPase region of Sup35 and showed that in most cases, they were incompatible with the [*PSI*^+^] prion. In rare cases, viable clones were selected that arose from cells that had lost the [*PSI*^+^] prion and retained the mutant allele, or, alternatively, that had lost the mutant allele due to recombination with *SUP35WT* but retained the prion. We concluded that the [*PSI*^+^] prion is only maintained in the presence of the *SUP35WT* allele. There are two alternative explanations for these results: (1) the mutations impair the ability of Sup35 to be prionized; (2) the mutations disrupt the GTPase activity of Sup35 proteins, which are still able to maintain yeast cell viability in [*psi*^−^] cells but not in [*PSI*^+^], where the available mutant Sup35 is depleted due to incorporation into prion aggregates.

**Figure 7:**
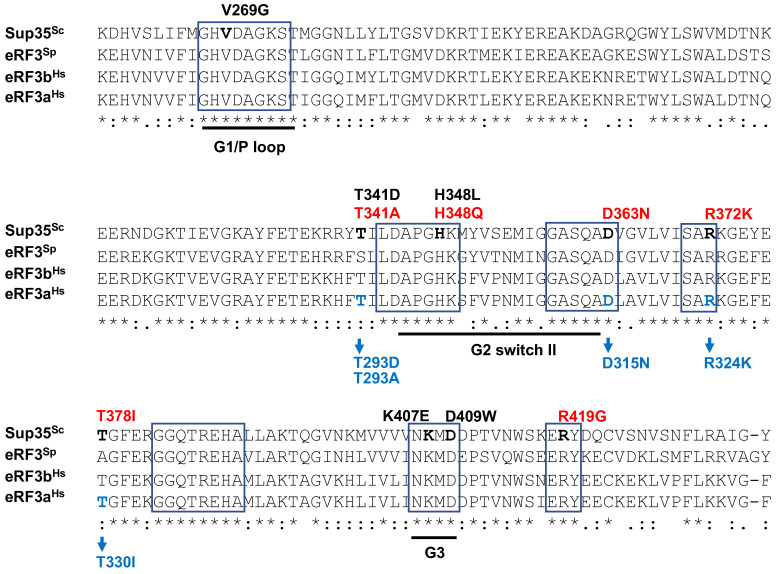
Multiple sequence alignment of *S. cerevisiae* Sup35 (aa 258–436), *S. pombe* eRF3 (aa 236–415), *H. sapiens* eRF3b (aa 201–379), and eRF3a (aa 210–388). The symbols (*), (:), and (.) indicate positions in the alignment that have fully, moderately, and weakly conserved residues, respectively. Designations of GTP-binding motifs (G1, G2, and G3) and Switch II regions are from [34]. Only invariant residue blocks where mutations were situated are shown. Lethal mutations are marked by black (V269G, H348L, K407E, D609W from [33]; T341D from [17]). Mutations with decreased GTPase activity are shown in red (H348Q, R419G from [33]; T341A from [17]; D363N, R372K, T378I—this work). Positions of mutations in yeast Sup35 correspond to the following positions in eRF3a: T341 to T293; D363 to D315; R372 to R324; T378 to T330 (shown in blue).

All the mutations studied (D363N, R372K, T378I) are located in the proximal part of Sup35C, which is highly conservative between different species (Figure 7). Multiple sequence alignment of *S. cerevisiae Sup35* (aa 258–436), *S. pombe* eRF3 (aa 236–415), *H. sapiens* eRF3b (aa 201–379) and eRF3a (aa 210–388) allowed us to identify aa residues of human eRF3a (GSPT1) corresponding to *sup35-m* mutations of *S. cerevisiae* and use them to measure the GTPase activity of mutated proteins. The following mutations were studied: T293D and T293A (corresponding to mutations T341D and T341A described previously [17,30]; D315N (corresponding to *sup35-25*); R324K (corresponding to *sup35-228*); T330I (corresponding to *sup35-10*). All these residues are located in the proximity of the G2 Switch II region in the GTPase fold of Sup35 (Figure 7). Mutations within the G1, G2, or G3 regions have previously been shown to be lethal (V269G, H348L, K407E, D609W from [33]). Here, we studied mutations localized in close proximity to the G2 region and they were found to disrupt the GTPase activity. Unlike the other known mutations with impaired GTPase activity, those studied here were obtained as spontaneous mutations that are viable under normal conditions, suggesting that the release factor activity is still tolerable in such mutants. However, the mutations may cause increased sensitivity to the factors that add an additional strain on the translational machinery, such as the presence of [*PSI*^+^], resulting in inability to propagate the prion despite the mutant proteins being able to form aggregates and co-aggregate with the native Sup35. According to the data obtained, the following amino acid substitutions: T293D, D315N, R324K, T330I, lead to a decrease in the GTPase activity of the corresponding human eRF3 proteins (Figure 6).

Previously, it was proposed that the T341 replacement in yeast Sup35 protein directly influences the properties of a prion [*PSI*^+^] [30]. Our data clearly show that the lethality of *sup35-m* mutations in combination with the [*PSI*^+^] prion is explained by defects in the GTPase activity of mutated proteins. Such low activity of mutant Sup35 combined with aggregation of Sup35 due to the presence of the [*PSI*^+^] prion is not sufficient to keep the viability of yeast cells.

## 4. Materials and Methods

### 4.1. Strains, Cultivation, and Microscopy

The yeast strains used in this study are listed in Table 1. All strains, except BY4742, contain deletion of the chromosomal *SUP35* gene compensated by a centromeric *URA3* plasmid containing *SUP35*, which is either pRSU2 [12] or pYCH-U2 [35]. Strain 10-7A-D832 contains a variant of [*PSI*^+^] which is phenotypically strong. *Escherichia coli* strain DH5α [36] was used for plasmid selection, maintenance and amplification. Derivatives from *E. coli* BL21(DE3) [37] were used to produce recombinant proteins (see below). Standard yeast and bacterial media with minor modifications were used [38,39]. YPGly medium is the same as YEPD, but contains 2.4% (*v*/*v*) of glycerol instead of glucose. For the plasmid shuffling experiments, cells containing two plasmids, one with *LEU2* marker and another with *URA3*, were plated onto the 5-FOA medium [38]. The resulting colonies were Ura^−^Leu^+^ due to the loss of the *URA3* plasmid, as 5-fluoroorotic acid counter-selects against *URA3*. For color selection, 1/4YEPD medium was used [40]. Yeast strains were grown at 30 °C or at 26 °C if they carried *sup35-m* mutations; *E. coli* strains were grown at 37 °C.

The transformation of yeast cells was performed according to the published protocol [43] with minor modifications. For the microscopy, cells were cultivated in liquid media to the logarithmic phase (OD_600_ = 0.6); then, 0.5 mL of culture was gently pelleted (1150 rcf, 1 min) and resuspended in 10 μL of liquid media with 5–10% glycerol. Fluorescence was analyzed using a AxioScope.A1 wide-field fluorescence microscope (Zeiss, Oberkochen, Germany). Images were taken with a QIClick-F-CLR-12 (QImaging, Surrey, BC, Canada) or Axiocam 506 color camera (Zeiss, Oberkochen, Germany) using QCAPTURE PRO 7 or ZEN 3.4 (blue edition) software, respectively.

### 4.2. Plasmids

Plasmids pRSU1P-10 and pRSU1P-25 with missense mutations in the *SUP35* Peterhof allele (designated as *SUP35P*) [12], as well as pRSU1B-228 with a mutation in the reference allele (designated as *SUP35B*) [10], were obtained previously. Plasmids pRSU1B-10, pRSU1B-25, and pRSU1P-228 containing the same mutations on opposite backgrounds were obtained as follows: pRSU1P-10, pRSU1P-25, and pRSU1B-228 were digested using Ksp22I and Mph1103I endonucleases. Ligation of the obtained fragments (1094 bp) from pRSU1P-10 and pRSU1P-25 into the backbone of pRSU1B-228 (9610 bp) yielded pRSU1B-10 and pRSU1B-25, respectively. Ligation of a similar fragment of pRSU1B-228 into the backbone of pRSU1P-25 yielded pRSU1P-228. All mutations analyzed in the study are listed in Table 2.

Plasmid pRS316-pCUP-SUP35NM-GFP (pRS316CNMG [44]) and a series of constructs derived from this plasmid, pRS316-pCUP-SUP35-GFP (bearing the *SUP35B* or *SUP35P* allele regulated by the copper-inducible *CUP1* promoter), pRS316-pCUP-sup35-10-GFP, and pRS316-pCUP-sup35-228-GFP, were used to induce the [*PSI*^+^] prion in the yeast cells. pRS316-pCUP-SUP35-GFP was obtained by replacing the *SUP35NM* fragment by the *SUP35* gene (*SUP35B* or *SUP35P* allele) at BamHI and SacII sites. The remaining constructions were obtained by cloning fragments of the corresponding *SUP35* alleles into the pRS316-pCUP-SUP35-GFP plasmid at AarI sites. pRS316-pCUP-GFP (pRS316CG [44]) was used as expression control.

Plasmids pET23_eRF1-6xHis (described in [45]) and pET-6xHis-SUMO-eRF3a (described in [46]) gifted from Elena Z. Alkalaeva (Engelhardt Institute of Molecular Biology, The Russian Academy of Sciences, Moscow), were used for recombinant human His-tagged eRF1 and His-SUMO-tagged eRF3a proteins production in bacteria. By site-directed mutagenesis of the *GSPT1* gene from pET-6xHis-SUMO-eRF3a plasmid, we obtained a series of expression vectors used for the same purposes.

Mutagenesis of human *GSPT1* was performed to generate constructs that were then used to produce recombinant eRF3a proteins in bacterial cells. The following mutations in eRF3a were introduced: D315N, T330I, R324K, which correspond to yeast *sup25-10*, *sup25-25* and *sup25-228* mutations, respectively (see Table 2); as well as T293A, and T293D substitutions similar to the T341A and T341D mutations described before [17]. The PCR template used was the pET-6xHis-SUMO-eRF3a plasmid and the primers are listed in Table 3. The template was fully amplified using AccuPrime Pfx DNA polymerase (Invitrogen, Thermo Fisher Scientific, Waltham, MA, USA). After PCR, the DNA mixture was digested with DpnI to remove the original template and was then used to transform the bacteria.

For the Sup35 protein purification, we obtained a series of expression vectors based on the pET-20b-SUP35NM-(His)_6_ plasmid [47], containing the full-length *SUP35* wild-type gene, or its Peterhof variant, and the *SUP35* gene with D363N (*sup35-10*), T378I (*sup35-25*), and R372K (*sup35-228*) substitutions. The pET20b-SUP35 vector used for the production of the wild-type Sup35 protein was obtained by subcloning a small PstI-PstI fragment from pET21b-ySUP35 [48] in place of an analogous fragment of pET-20b-SUP35NM-(His)_6_. The remaining constructions were obtained by cloning fragments of the corresponding *SUP35* alleles from pRSU1-based vectors into the pET20b-SUP35 plasmid at AarI sites.

### 4.3. [*PSI*^+^] Induction

Plasmids bearing the *GFP*-tagged *SUP35* gene variants or the *GFP* gene under control of the *CUP1* promoter were used. The resulting transformants were grown in a liquid selective medium at 30 °C until OD_600_ was 0.1–0.2, after which CuSO_4_ was added to a final concentration of 50 μM; after that, cells were grown for another 24 h. Then, equal aliquots of cells were plated on 1/4YEPD medium, grown for 3–4 days, and the number of white (Ade^+^) colonies was counted. The remaining cells were collected for analysis of aggregates by fluorescence microscopy. Presence of the [*PSI*^+^] prion was determined by the white or pink color on the 1/4YEPD medium and Ade^+^ phenotype, both caused by the suppression of the *ade1-14* allele.

### 4.4. Semi-Denaturing Detergent Agarose Gel Electrophoresis (SDD-AGE)

SDD-AGE [49] was used for the analysis of Sup35 amyloid aggregates, followed by capillary transfer onto PVDF membrane [49,50] and Western blot hybridization [39]. The mix (1:1) of rabbit polyclonal anti-Sup35 (SE4290) and anti-Sup35N (SE4291) [10] antibodies was used to detect Sup35. Defatted powdered milk in Tween Tris-buffered saline (TTBS) was used for PVDF membrane blocking (5% *w*/*v*) and as a diluent buffer for antibodies. Yeast protein lysates were prepared as previously described [51]. Usually, 30–50 μg of total protein per lane was loaded depending on the experiment. To determine the protein concentration in the lysates, we performed a Bradford assay using Quick Start™Bradford 1x Dye Reagent (Bio-Rad, Hercules, CA, USA) according to the manufacturer’s recommendations. The standard protocol for the 250 μL microplate assay was used.

### 4.5. Purification of Proteins

His-tagged Sup35 proteins used for fibril assembly were produced in *E. coli* strain NiCo21(DE3) (New England BioLabs (Ipswich, MA, USA)) and purified under native conditions as described previously [14].

For the GTPase assay, 80S yeast ribosomal particles were purified from yeast BY4742 cell lysate as described previously [52,53]. Human His-tagged eRF1 and His-SUMO-tagged eRF3a proteins were produced in *E. coli* strain BL21(DE3)pLysS (Invitrogen, Waltham, MA, USA). Recombinant proteins were purified using Ni-NTA agarose and ion-exchange chromatography as described previously [46,52,54].

### 4.6. Assembly of Sup35p into Protein Fibrils

Recombinant Sup35 proteins were adjusted to a concentration of 2 mg/mL and used to prepare filaments (see [14]). For aggregation in the presence of pre-existing fibrils, Sup35NM fibrils (0.5 mg/mL) were added to the protein solutions in a 1:5 ratio. The fibrils were pretreated on a sonicator SONOPULS HD 2070 (BANDELIN electronic GmbH & Co. KG, Berlin, Germany) for 20 s at 50% power. Aggregation was carried out at 4 °C with constant stirring on a rotator. SDD-PAGE was performed to detect Sup35 aggregates. For that, samples before and after aggregation of equal volume were taken and loading buffer was added [55]. Then, one sample was boiled (100 °C) for 5 min, and the other was incubated at 25 °C. As a result of boiling in the presence of SDS, the protein aggregates were denatured to monomers and could pass through the pores of the polyacrylamide gel, whereas in unboiled samples, the aggregates were preserved and could not be separated in the gel. The resulting gel was stained with Coomassie. Protein aggregation was determined by the difference in band thickness in the boiled and unboiled samples.

### 4.7. Electron Microscopy

For fibril visualization, a Jeol JEM-1400 transmission electron microscope (JEOL Ltd., Peabody, MA, USA) was used. Samples were prepared by applying 10 μL of the aggregates solution on a formvar-coated grid for 60 s, followed by washing with distilled water. Then, the samples were stained with the 1% (*w*/*v*) uranyl acetate for 60 s. The excess of the dye was removed with incubation in distilled water for 30 s.

### 4.8. Assay for GTPase Activity

To measure the GTPase activity of eRF3, we prepared the following mixture in a final reaction volume of 10 μL: 80S ribosomes, eRF1 and eRF3a—5 pmol of each, 23.5 mM Tris-HCl pH 7.5, 35 mM NH_4_Cl, 10 mM MgCl_2_, 0.5 mM GTP. After incubation for 20 min at 37 °C, the amount of released phosphate was estimated with Malachite Green Phosphate Assay (Sigma-Aldrich, St. Louis, MI, USA), according to the manufacturer’s protocol.

### 4.9. Statistical Analysis

Statistical processing of the frequencies of [*PSI*^+^] occurrence in yeast cells was performed using the Wilcoxon rank-sum test adjusted for multiple comparisons (Holm’s method). To evaluate the differences in phosphate released between eRF3a proteins, Welch’s *t*-test and the Wilcoxon rank-sum test adjusted for multiple comparisons (Benjamini–Hochberg correction) were performed. All calculations were carried out in R for statistical analysis [56]. Differences were considered statistically significant at the *p* < 0.05 level.

## Figures and Tables

**Table 1:** Yeast strains used in the study.

Strain	Genotype	Reference
7A-D832	*MATαade1-14 his7-1 leu2-3,112 lys2-739 trp1-289 ura3-52 SUP35::TRP1* [pYCH-U2] [*PIN*^+^] [*psi*^−^]	[41]
10-7A-D832	*MATαade1-14 his7-1 leu2-3,112 lys2-739 trp1-289 ura3-52 SUP35::TRP1* [pYCH-U2] [*PIN*^+^] [*PSI*^+^]	[41]
U-14-D1690	*MATαade1-14 trp1-289 his3-Δ200 lys2 ura3-52 leu2-3,112 SUP35::HIS3MX* [pRSU2] [*PIN+*] [*psi*^−^]	[9]
BY4742	*MATα his3Δ1 leu2Δ0 lys2Δ0 ura3Δ0* [*PIN*^+^] [*psi*^−^]	[42] (A gift from Youri I. Pavlov)

**Table 2:** The characteristic of *SUP35* alleles used in this study.

*SUP35* Allele	Nucleotide Position in *SUP35*	Nucleotide Position in *hGSPT1*	Mutation	Amino Acid Position in Sup35	Amino Acid Position in hGSPT1 (eRF3a)	Codon Substitution
*sup35-10*	1087	931	G → A	363	315	Asp → Asn
*sup35-25*	1133	977	C → T	378	330	Thr → Ile
*sup35-228*	1115	959	C → T	372	324	Arg → Lys

**Table 3:** Primers used for human *GSPT1* mutagenesis.

Primer	Sequence 5′–3′ *
10-eRF3a-human-F	GTGCCTCTCAAGCTAATTTGGCTGTGCTG
10-eRF3a-human-R	CAGCACAGCCAAATTAGCTTGAGAGGCAC
25-eRF3a-human-F	GAAAGGAGAGTTTGAAATTGGATTTGAAAAAGGAG
25-eRF3a-human-R	CTCCTTTTTCAAATCCAATTTCAAACTCTCCTTTC
228-eRF3a-human-F	CTGGTAATCTCAGCCAAGAAAGGAGAGTTTG
228-eRF3a-human-R	CAAACTCTCCTTTCTTGGCTGAGATTACCAG
T341A-eRF3a-human-F	GAAAAGAAGCATTTCGCAATTCTAGATGCCCCTG
T341A-eRF3a-human-R	CAGGGGCATCTAGAATTGCGAAATGCTTCTTTTC
T341D-eRF3a-human-F	ACCGAAAAGAAGCATTTCGACATTCTAGATGCCCCTGG
T341D-eRF3a-human-R	CCAGGGGCATCTAGAATGTCGAAATGCTTCTTTTCGGT

* The nucleotide substitutions are underlined.

## Data Availability

The data used and analyzed during the study are available from the corresponding author on reasonable request.

## Abbreviations

The following abbreviations are used in this manuscript:

*sup35-n*	*sup35* nonsense mutations
*sup35-m*	*sup35* missense mutations
*sup45-n*	*sup45* nonsense mutations
*sup45-m*	*sup45* missense mutations
PGC	Peterhof Genetic Collection
SDD-AGE	Semi-Denaturing Detergent Agarose Gel Electrophoresis
PFD	Prion-Forming Domain
